# The Way to Kill Waiting-Lists, and How to Pay for Additional Upkeep

**Published:** 1917-11-03

**Authors:** 


### THE ORGANISATION OF SECTIONAL SUBSCRIPTIONS.

### The Way to Kill Waiting-Lists, and How to Pay for Additional Upkeep.

We lately touched on the lighter side of Mr. Leonard Rea's appeals on behalf of the King Edward VII. Hospital, Cardiff, where, as the hospital authorities know, the problem of the waiting-list is, and has been, to put it bluntly, a scandal. To make the public realise this a small and generous group of men under Colonel Bruce-Vaughan's able leadership has spared no pains, and thinking men are beginning to work behind and with him. A symptom of this was the recent letter, signed Howell Rees, in the *South Wales Echo*. Dr. Rees remarked in the course of a convincing argument that Sir Henry Burdett had already warned the men of the South Wales coalfield and the shipping industry of the need for 1,500 to 2,000 more beds, distributed, the writer added, between Swansea and Newport in the proportion of 300 more apiece, and for the rest looking to Cardiff for 600 more, and to the smaller urban and cottage hospitals for a further 300 or 400. Sir Henry Burdett's series of Reports on the Hospitals of the United Kingdom was in part designed to start a campaign in furtherance of increased hospital accommodation, though, as an old hand, he limited himself to an exposure of the need for rebuilding and reconstruction in individual instances. The criticism which was evoked by his reports, the (occasional) frantic denials of anything amiss, and the reiterated plea of want of funds were the familiar signs which showed that he had put his finger on the weak spot. The criticism soon blew over; and the noise, as it were, of many protesting waters soon gave way to the cheerful clink of builders' hammers. At Leeds, at Chester, at Cardiff (which last needed a jog perhaps least of all) new buildings began to rise, the money that *could* not be raised began to flow in, and the point was won. Soon after the hospitals had begun thus to stir themselves the war came, and with it the loiterers, for there were a few still, were simply swept aside as negligible. Desire to imitate achieved what a friendly criticism here and there had failed to accomplish. That the weakness of the voluntary system was its gross lack of beds became a commonplace, while in war, as before it, Cardiff continued to hold a leading place in the van.

The work at Cardiff, however, as Dr. Howell Rees is fully aware, is far from complete. But its urgency is admitted, and its extent is now beginning to be known. Let us, in the interest of other hospital centres, consider Dr. Rees's proposals for footing the necessarily heavy bill. We take his proposals as a text, since other hospitals are in a similar position to that over which General Lee and Colonel Bruce-Vaughan preside. Besides, a definite policy for one set of circumstances in one place is always suggestive in a similar set elsewhere.

The war at least has taught us all neither to boast nor to be afraid of large figures. Dr. Howell Rees begins, then, by asking for £150,000, not by any means as a total, but merely as a modest contribution from the other rich men of South Wales, who must have learned something from the generous public spirit of their leaders, Sir W. J. Thomas and Sir Edward Nicholl. With this nucleus, Dr. Howell Rees believes that a thousand beds in Cardiff could be completed. It will be remembered that steps in the right direction have already begun to mature.

How about maintenance? The flaw in the present system of income-raising in South Wales is flatly stated to be the haphazard way in which the workmen's subscriptions are organised. Even Wales, it seems, does not organise perfection in its sleep. We learn—and the example of Newcastle and Leicester is mentioned to point the moral—that "some of the largest steam collieries only contribute [their] tens," while contributions from some of the smaller collieries deserve to be regarded as their "hundreds." It is proposed, then, to organise the weekly contributions not only in relation to the wages of the men, but *also in relation to each other*. There is to be a general levelling up. At least there should be. Of the men's willingness we say nothing. Has it not been proved a thousand times that willingness is only waiting to be shown the way? The question of asking that the weekly sum may be raised to twopence among the suitable classes of men which are to be found in every colliery is frankly considered. We believe that from weekly pennies at present King Edward VII.'s Hospital in Cardiff still receives only some £6,000 a year, and Swansea Hospital merely £9,000. We wish again to rub in the fact that from similar weekly collections Leicester Infirmary receives £15,000, and Newcastle £25,000 a year. It is, indeed, an odious comparison.

The man to organise the reconstruction of subscriptions, which is a necessary part of any reconstruction of bricks and mortar, has not to be found. He is already there. Mr. R. Evans, the secretary of the Cardiff and District Hospital Fund, only wants a lead. He is always ready for new coalfields to conquer! The coal industry, however, is a pit of many mansions. There are the owners, who should put up the buildings. There are "the men," who should maintain them. Who are "the men"? Not one class, but many. They are the miners, the foremen, the managers, the clerical staff, the shippers, the very tradespeople connected with the winning and conveyance of coal. At least seven classes. There is, too, another class, the most elusive of all—the indistinguishable, but ubiquitous shareholder. Why should the owners be the only class in this industry to follow the lead of the colliers and to organise their hospital gifts? The big maintenance bill of a modern hospital

THE ORGANISATION OF SECTIONAL SUBSCRIPTIONS—(*continued*).

needs organised subscriptions *from every class which it serves.* We have long cried this to unheeding ears. No matter; what prevision, experience, argument could not teach, stern necessity has begun to din into them. At York subscriptions from farmers are being organised, or about to be so. At North Staffordshire the big employers are being organised so that they may agree to exchange a haphazard cheque for a steady annual gift computed at one shilling for each person in their employment. A humble beginning, but a good one. And, as Mr. Basil Rhodes explained in THE HOSPITAL of October 20, the local employees' associations have been eager and willing to fall into the new plan. At last, in fact, the sectional organisation of subscriptions is beginning to be understood as the solution of the maintenance problem.

We bring these different examples from York, from the Midlands, and now from South Wales together that the principle behind them may be apparent and applied to every hospital centre where there are brains enough to seize the basis of the new policy, and determination enough to apply it. Flag days make a flare. House-to-house collections are the shift of desperation. Bazaars, concerts, *café chantants*, pound days are at best expedients, and very costly in time and tact. *There is no financial policy in these.* They come and go, but the sectional organisation of subscriptions from every class can continue for ever and ever. By this means the twin problem of how to abolish waiting-lists by fresh building and how to pay for the consequent increased maintenance can be solved. Have our hospital men sufficient intelligence and grit to apply it?

